# Effects of Transcranial Electrical Stimulation on Intermuscular Coherence in WuShu Sprint and KAN-Based EMG–Performance Function Fitting

**DOI:** 10.3390/s25196241

**Published:** 2025-10-09

**Authors:** Lan Li, Haojie Li, Qianqian Fan

**Affiliations:** 1 Physical Education Department, Woosuk University, Wanju-gun 55338, Republic of Korea; 13032425419@163.com; 2 School of Exercise and Health, Shanghai University of Sport, Shanghai 200438, China; 202121070037@mail.bnu.edu.cn

**Keywords:** transcranial electrical stimulation, intermuscular coherence, neural network modeling, sprint biomechanics, electromyography

## Abstract

Objective: The aim of this study was to examine how transcranial electrical stimulation (tES) modulates intermuscular coherence (IMC) in sprinters and develop an interpretable neural network model for performance prediction. Methods: Thirty elite sprinters completed a randomized crossover trial involving three tES conditions: motor cortex stimulation (C1/C2), prefrontal stimulation (F3), and sham. Sprint performance metrics (0–100 m phase analysis) and lower-limb sEMG signals were collected. A Kolmogorov–Arnold Network (KAN) was trained to decode neuromuscular coordination–sprint performance relationships using IMC and time–frequency sEMG features. Results: Motor cortex tDCS increased 30–60 m sprint velocity by 2.2% versus sham (*p* < 0.05, η^2^ = 0.25). γ-band IMC in key muscle pairs (rectus femoris–biceps femoris, tibialis anterior–gastrocnemius) significantly heightened under motor cortex stimulation (F > 4.2, *p* < 0.03). The KAN model achieved high predictive accuracy (R^2^ = 0.83) through cross-validation, with derived symbolic equations mapping neuromuscular features to performance. Conclusions: Targeted tDCS enhances neuromuscular coordination and sprint velocity, while KAN provides a transparent framework for performance modeling in elite sports.

## 1. Introduction

In the neurobiochemical system of competitive sports, the high specificity of sprint performance depends on the precise regulatory efficiency of the central nervous system over skeletal muscle groups [1]. As a key bioelectrical indicator for quantifying this neuromuscular coordination, intermuscular coherence (IMC) directly reflects the level of cluster control of motor units by upper spinal neurons by analyzing the phase synchrony of surface electromyography (sEMG) signals in the gamma band (30–60 Hz) [2,3]. Studies have shown that athletes exhibit significantly enhanced tibialis anterior-gastrocnemius IMC values during the acceleration phase (Cohen’s d = 1.32, *p* < 0.01), and this coordination pattern can reduce antagonist muscle co-activation losses by 18.7 ± 3.2%, directly correlating with the biomechanical benefits of stride length–cadence optimization [4]. However, traditional training methods for regulating IMC are subject to a neuroadaptive ceiling effect, necessitating the intervention of novel neurostimulation technologies to overcome this limitation.

Transcranial electrical stimulation (tES) modulates motor cortex excitability through polarized currents and has been shown to remodel synaptic efficacy in the corticospinal pathway. Recent studies have revealed that anodal tDCS applied to the primary motor cortex (M1 region) can increase the slope of the recruitment curve of the corticospinal tract by 22% while reducing the resting period of motor evoked potentials (MEPs) [5]. Such changes in neural plasticity should theoretically directly influence the synchronized discharge patterns transmitted down to the muscles. However, existing studies have primarily focused on macro-level performance indicators [6] and have not yet established a three-tier causal chain linking tES parameters (current density and stimulation polarity) to IMC characteristics and motor performance. Notably, theta burst transcranial alternating current stimulation (tACS) may enhance γ-β-band IMC through the entrainment effect of brain waves, a hypothesis that requires experimental validation [7].

The current modeling of IMC–performance relationships faces two major challenges: first, the IMC features extracted by traditional frequency-domain coherence analysis (such as Morlet wavelet transform) exhibit significant nonlinear coupling with dynamic parameters (such as peak ground reaction force); second, conventional machine learning methods (such as support vector regression) cannot simultaneously meet the requirements for prediction accuracy and model interpretability. The KAN achieves this through adaptive activation function topology optimization, converting hidden-layer weights into explicit differential equations. In vestibular–ocular system modeling, it has achieved a variance explanation rate of 92.7% (R^2^ = 0.927) while maintaining physiological interpretability of parameters [8]. This white-box modeling advantage provides a new tool for analyzing the transmission equations of tES–IMC–performance.

This study innovatively integrates neurostimulation technology with computational modeling methods to address two core questions: (1) Can tES intervention optimize the temporal coordination of the sprinting kinetic chain by enhancing γ-β-band IMC? (2) Can the EMG–performance function based on KAN quantitatively predict performance gains under different tES parameters? The research findings will establish the first quantitative predictive model linking “electrical stimulation parameters–neural synchrony–motor output,” providing a theoretical basis for developing personalized neural enhancement protocols and advancing competitive training into a new paradigm of precision neural modulation.

## 2. Methods

### 2.1. Participants

A priori statistical power analysis was conducted using G*Power 3.1.9 software (F-test family, repeated measures analysis of variance), with the effect size set as ES = 0.2 (based on pre-experiment data), significance level α = 0.05, statistical power 0.8, and intra-class correlation 0.5. The calculated minimum sample size was N = 20. Considering a 10% sample attrition rate, 30 national level-2-or-above Wushu Athlete (15 males and 15 females) were ultimately recruited, with the actual statistical power reaching 0.83 (post hoc verification). Baseline data of the participants, including core indicators such as age, training years, 100 m personal best (PB) time, and body fat percentage, are shown in Table 1. The research protocol was approved by the Ethics Committee of Shanghai University of Sport (No. SUS20250411), and all participants signed informed consent forms, retaining the right to withdraw unconditionally.

Inclusion and Exclusion Criteria: Participants were required to have engaged in continuous professional sprint training for ≥5 years, ranked among the top 8 in provincial-level competitions within the past three years, and been rated as “high-intensity active” (MET-min/week ≥ 3000) by the International Physical Activity Questionnaire (IPAQ-SF). Lower-limb muscle symmetry was quantified using the Biodex system (bilateral quadriceps peak torque difference ≤ 15%). Individuals with neuromuscular diseases, tES contraindications (such as epilepsy or intracranial implants), acute lower-limb injuries in the past six months (Lysholm score < 90), and a history of neuroactive drug use were excluded. Additionally, the Morningness–Eveningness Questionnaire was used to screen for circadian rhythm stability (score ≥ 30), and daily caffeine intake was restricted to ≤200 mg to control potential confounding factors interfering with neuroregulatory effects.

**Table 1:** Baseline characteristics of participants (M ± SD).

Indicator	Male (*n* = 15)	Female (*n* = 15)
Age (years)	22.1 ± 1.8	22.5 ± 2.1
Training years	5.9 ± 1.2	6.2 ± 1.4
100 m PB (seconds)	11.02 ± 0.15	12.07 ± 0.18
Body fat percentage (%)	10.3 ± 1.1	17.5 ± 1.3

### 2.2. Experimental Design

This study adopted a randomized repeated-measures design, in which participants received three intervention conditions in sequence: anodal tDCS (2 mA, 20 min) targeting the primary motor cortex (C3/C4 areas, 10–20 system positioning) and dorsolateral prefrontal cortex (F3/F4 areas), respectively, and sham stimulation (gradually increasing to 0.5 mA over 30 s before turning off the current). A 14-day washout period was set between interventions, during which high-intensity training and neuroregulatory techniques were prohibited. All tests were conducted uniformly between 08:00 and 10:00, with a standardized warm-up (including 3 sets of 30 m sprints at 80% maximum speed, dynamic stretching, and reaction training) performed 1 h before the test to control the interference of circadian rhythm and warm-up intensity on the results.

**Figure 1:**
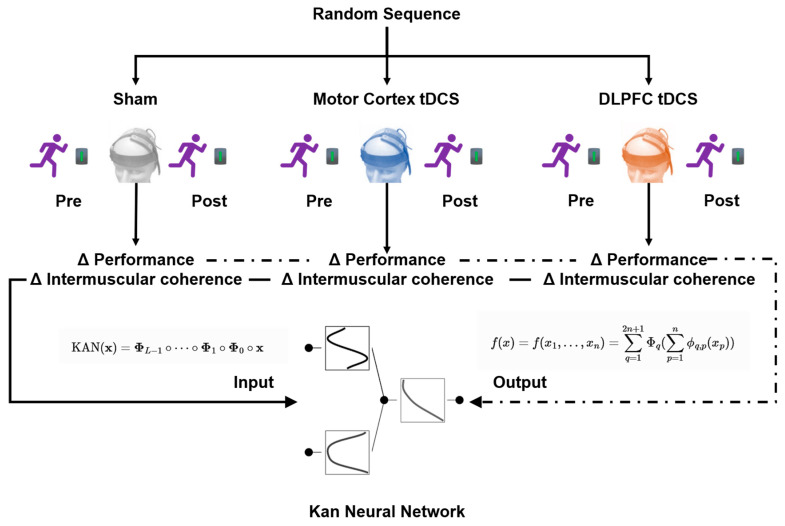
Flowchart of experimental condition allocation.

The intervention process included baseline testing (measuring 100 m performance and intermuscular coherence of target muscle groups 24 h before intervention), generation of randomized sequences (using Randomization.com software V1.5), and double-blind stimulation implementation (NeuroStim 2 device, 5 × 5 cm electrodes). For the motor cortex group, the anode was placed on the C3/C4 area (contralateral supraorbital cathode), while for the dorsolateral prefrontal cortex (DLPFC) group, the anode was placed on the F3/F4 area (ipsilateral arm cathode). Within 5 min after stimulation, a 100 m sprint was conducted, with motor performance and intermuscular coherence data recorded simultaneously (Figure 1).

### 2.3. Anodal tDCS Stimulation Protocol

**Figure 2:**
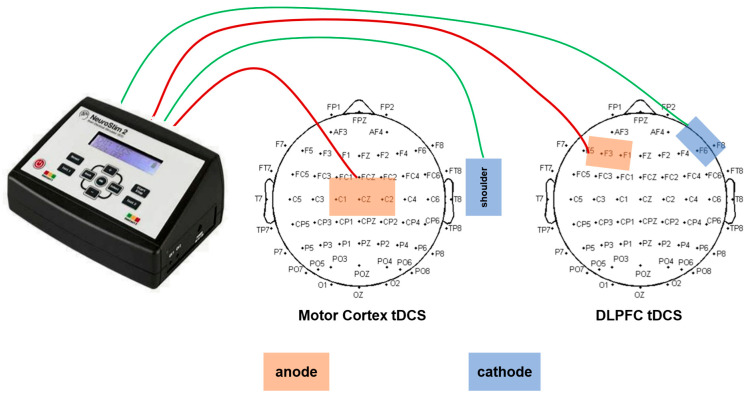
tDCS anode and cathode placement.

A double-blind randomized design was used, with interventions delivered by a NeuroStim 2 transcranial direct current stimulator (Medina Tebgostar, Iran) via 7 × 5 cm carbon rubber electrodes (sponges impregnated with physiological saline, current density 0.08 mA/cm^2^). Electrode positioning was based on the international 10–20 EEG system, with target areas precisely calibrated using a 64-lead EEG cap: the anode for motor cortex stimulation was placed on the C1/C2 area (corresponding to the lower-limb motor cortex), and the cathode was fixed on the ipsilateral deltoid muscle; the anode for DLPFC stimulation was placed on the F3 area (dorsolateral prefrontal cortex), and the cathode was placed on the contralateral supraorbital area. Although electrode positions were targeted to modulate specific cortical regions, it is recognized that tDCS current distribution extends beyond the anode site to broader cortical/subcortical networks, and is critically shaped by both electrode placements [9]. The sham stimulation group used the same electrode layout, with the current gradually increasing to 0.5 mA over 30 s before being turned off, maintaining electrode touch but without effective neuroregulation (Figure 2).

Stimulation parameters were set as 2 mA constant current output for 20 min, including 30 s ramp-up/ramp-down periods. All participants were informed that the experiment aimed to “compare different neuroregulation modes,” and the existence of sham stimulation was not explicitly disclosed to avoid expectancy effects.

### 2.4. The 100 M Performance Test

The 100 m sprint performance was recorded by the Optojump Next infrared timing system (Microgate, Bolzano, Italy), which consists of 32 pairs of gratings with a spacing of 0.5 m (sampling rate 1000 Hz), covering the entire distance from the starting line to the finish line. Before the test, participants completed a standardized warm-up (including 3 sets of 30 m acceleration runs at 80% intensity and dynamic stretching), and after a 20 min rest, they performed a single all-out sprint. A standing start was used, and the timing was divided into segments: 0–30 m (acceleration phase), 30–60 m (speed maintenance phase), and 60–100 m (speed endurance phase) (precision ± 0.001 s). Step counts derived from Optojump foot-strike analysis averaged 17.3 ± 1.1 steps (0–30 m), 13.6 ± 0.9 steps (30–60 m), and 19.2 ± 1.3 steps (60–100 m) across participants. During the test, the surface electromyography system Delsys Trigno (manufactured by Delsys Inc., Natick, MA, USA) trigger signals were recorded simultaneously to ensure millisecond-level alignment of timing data with intermuscular coherence (IMC) analysis. Three tests were conducted at 48 h intervals, and the best result was used for analysis.

### 2.5. Electromyography Test and Intermuscular Coherence Analysis

**Figure 3:**
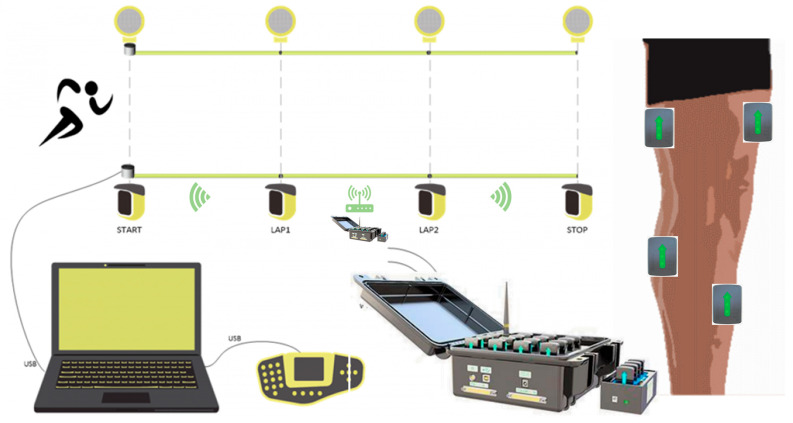
The 100 m performance test and EMG test equipment.

**Data Collection:** Electromyographic signals were recorded unilaterally on the right lower limb using a wireless surface electromyography system **Delsys Trigno** (manufactured by Delsys Inc., Natick, MA, USA). Electrodes were positioned over four key muscles, the rectus femoris (knee extension/hip flexion), biceps femoris (knee flexion/hip extension), lateral gastrocnemius (ankle plantarflexion), and tibialis anterior (ankle dorsiflexion), following SENIAM guidelines. Impedance was maintained at ≤5 kΩ, with a sampling rate of 1000 Hz and bandwidth of 20–500 Hz. Signal acquisition was synchronized with sprint phases using Optojump triggers to ensure temporal alignment between biomechanical events and neuromuscular activation patterns (Figure 3).

**Signal Processing**: Raw signals were processed by band-pass filtering (20–450 Hz), full-wave rectification, and 50 ms root mean square smoothing. Based on the trigger signals from the Optojump system, the time-domain features (integrated electromyography value iEMG, root mean square amplitude RMS) and frequency-domain features (median frequency MF) of each phase were extracted.

**Intermuscular Coherence Calculation**: The Halliday time–frequency coherence algorithm was used to calculate the coherence values of the rectus femoris–biceps femoris pair in the α (8–15 Hz), β (15–30 Hz), and γ (30–50 Hz) frequency bands:

$$\mathrm{Coherence}(f)=\frac{|S_{xy}(f){|}^{2}}{S_{xx}(f)\cdot S_{yy}(f)}$$

where $S_{xy}(f)$ is the cross-power spectral density, and $S_{xx}(f)$ and $S_{yy}(f)$ are the auto-power spectral densities. The significant coherence threshold was defined as 0.5; α-band coherence (associated with slow movements and isometric contractions) was interpreted as reflecting overall force coordination; β-band coherence (occurring during weak tonic contractions) was associated with stride frequency fine-tuning; and γ-band coherence (prominent during strong contractions like running) reflected explosive neural drive [10]. Data were segmented according to 0–30 m (acceleration phase), 30–60 m (speed maintenance phase), and 60–100 m (speed endurance phase) phases to analyze differences in neuromuscular coordination patterns across phases.

### 2.6. KAN Construction

This study constructed a parameterized model based on the Kolmogorov–Arnold representation theorem, which states that any continuous function $f:[0,1{]}^{n}\to R$ can be decomposed into a finite combination of univariate functions and additive operations:

$$f(x)=\sum_{q=1}^{2n+1}\Phi_{q}\left(\sum_{p=1}^{n}\phi_{q,\;p}(x_{p})\right)$$

where $\phi_{q,\;p}:[0,1]\to R$ and $\Phi_{q}:R\to R$ are continuous functions. In the KAN, these functions are parameterized by cubic B-spline basis functions $B_{i}^{3}(x)$, expressed as follows:

$$B_{i}^{3}(x)=\frac{(x-t_{i}{)}^{3}}{6h^{3}}\cdot I[t_{i}\leq x<t_{i+1}]+\frac{-3(x-t_{i}{)}^{3}+3h\;(x-t_{i}{)}^{2}+3h^{2}(x-t_{i})+h^{3}}{6h^{3}}\cdot I[t_{i+1}\leq x<t_{i+2}]$$

$$+\frac{3(x-t_{i+3}{)}^{3}-3h(x-t_{i+3}{)}^{2}+h^{3}}{6h^{3}}\cdot I[t_{i+2}\leq x<t_{i+3}]+\frac{(t_{i+4}-x{)}^{3}}{6h^{3}}\cdot I[t_{i+3}\leq x<t_{i+4}]$$

where $t_{i}$ is the knot vector and h is the knot spacing.

The network input layer includes multi-dimensional variables such as intermuscular coherence parameters and time–frequency-domain electromyographic features, with the output layer being the 100 m segmental average acceleration. A width configuration of [9,5,3,1] was adopted, and the mapping from layer l to layer l+1 is

$$x_{j}^{l+1}=\sum_{i=1}^{n_{l}}\Phi_{ij}^{l}(x_{i}^{l})$$

where $\Phi_{ij}^{l}(x)=\sum_{k=0}^{K}c_{ijk}^{l}B_{k}^{3}(x)$, $c_{ijk}^{l}$ are spline coefficients, and K=3 corresponds to the grid parameter.

The training set included 270 groups of data (30 people × 3 times × 3 gait phases), divided using three-fold cross-validation. The objective function combined prediction error and regularization constraints:

$$L\;(\Theta )=\frac{1}{N}\sum_{n=1}^{N}{\left(y_{n}-f(x_{n};\Theta )\right)}^{2}+\lambda \left(\mu_{1}\sum_{l,i,j}\|\Phi_{ij}^{l}{\|}_{1}+\mu_{2}\sum_{l,i,j}S(\Phi_{ij}^{l})\right)$$

where the entropy regularization term is defined as follows:

$$S(\Phi_{ij}^{l})=-\sum_{k=0}^{K}\frac{(c_{ijk}^{l}{)}^{2}}{\|\Phi_{ij}^{l}{\|}_{2}^{2}}log\left(\frac{(c_{ijk}^{l}{)}^{2}}{\|\Phi_{ij}^{l}{\|}_{2}^{2}}\right)$$

The phased optimization strategy was as follows:

1. Basic training: Involves 20 iterations of L-BFGS optimization ($\lambda =0.01,\mu_{1}=1,\mu_{2}=10$)
2. Symbolic compression: Involves 20 iterations of selecting optimal expressions from the predefined function library $F=\{x,x^{2},exp,log,sin\}$ to minimize the following expression:
  $$\hat{f}(x)=arg\;\underset{g\in G}{min}\left(\frac{1}{N}\sum_{n=1}^{N}{\left(y_{n}-g(x_{n})\right)}^{2}+\lambda_{c}C(g)\right)$$

Model performance was evaluated using the following indicators:

$$R^{2}=1-\frac{\sum_{n=1}^{N}{\left(y_{n}-{\hat{y}}_{n}\right)}^{2}}{\sum_{n=1}^{N}{\left(y_{n}-\bar{y}\right)}^{2}}$$

**Figure 4:**
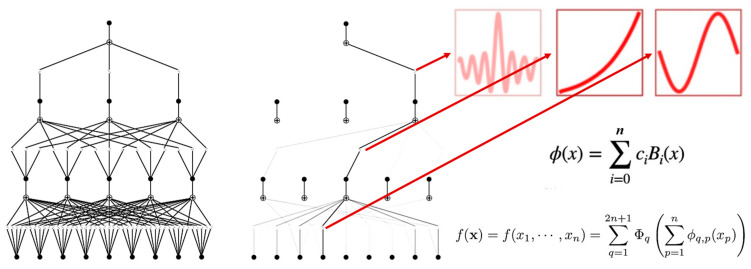
KAN architecture.

Key connection weights (|w|>0.2) were analyzed through topological visualization, and the final explicit equation revealed the cross-frequency-band coupling mechanism of electromyographic parameters on acceleration (Figure 4).

### 2.7. Statistical Analysis

Data analysis was performed using SPSS 26.0 (IBM, Armonk, NY, USA). First, the Shapiro–Wilk test was used to verify data normality (*p* > 0.05), with Q-Q plots assisting in evaluating distribution patterns. For intergroup differences among the three intervention conditions (motor cortex tDCS, DLPFC tDCS, sham stimulation), repeated-measures analysis of variance was conducted, with motor performance (100 m segmental speed) and intermuscular coherence (α/β/γ-band coherence values) as dependent variables. When Mauchly’s sphericity test indicated hypothesis violation (*p* < 0.05), Greenhouse–Geisser correction was used for degrees of freedom.

Significant main effects were followed by post hoc tests with Bonferroni correction. Within-group effect sizes were quantified by partial η^2^ (Partial Eta-Squared). All statistical tests used a two-tailed threshold of α = 0.05.

## 3. Results

### 3.1. Key Results of Sprint Performance (Table 2)

Repeated-measures ANOVA revealed a significant main effect of intervention conditions on sprint velocity during the 30–60 m phase (F(2,27)=4.62, p=0.019, $\eta_{p}^{2}=0.255$). Post hoc Bonferroni-corrected comparisons demonstrated that the motor cortex tDCS condition significantly outperformed the sham condition (p=0.017).

### 3.2. Key Results of Intermuscular Coherence (IMC) (Table 3)

Significant differences in IMC were observed during the 30–60 m phase across conditions, specifically the following:

- RA-BF Muscle Pair (γ-band): F(2,27)=6.11, p=0.005, $\eta_{p}^{2}=0.31$.
- TA-GL Muscle Pair (γ-band): F(2,27)=4.24, p=0.025, $\eta_{p}^{2}=0.24$.

Post hoc analyses indicated that motor cortex tDCS elicited significantly higher γ-band coherence compared to both sham (p<0.01) and DLPFC tDCS (p<0.05) conditions.

### 3.3. KAN Modeling

**Figure 5:**
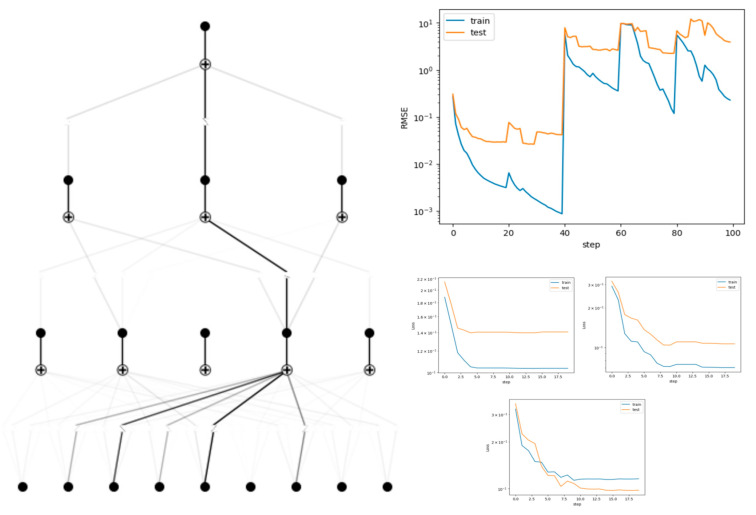
Trained and pruned models of the KAN, and training loss function curves.

1. Grid Parameter Optimization:

The model’s fitting performance was evaluated across grid parameters [3, 10, 20, 50, 100] with 20 training epochs. As illustrated in Figure 5, grid = 5 exhibited optimal fitting performance (minimum validation loss), balancing model complexity and generalizability.

**Figure 6:**
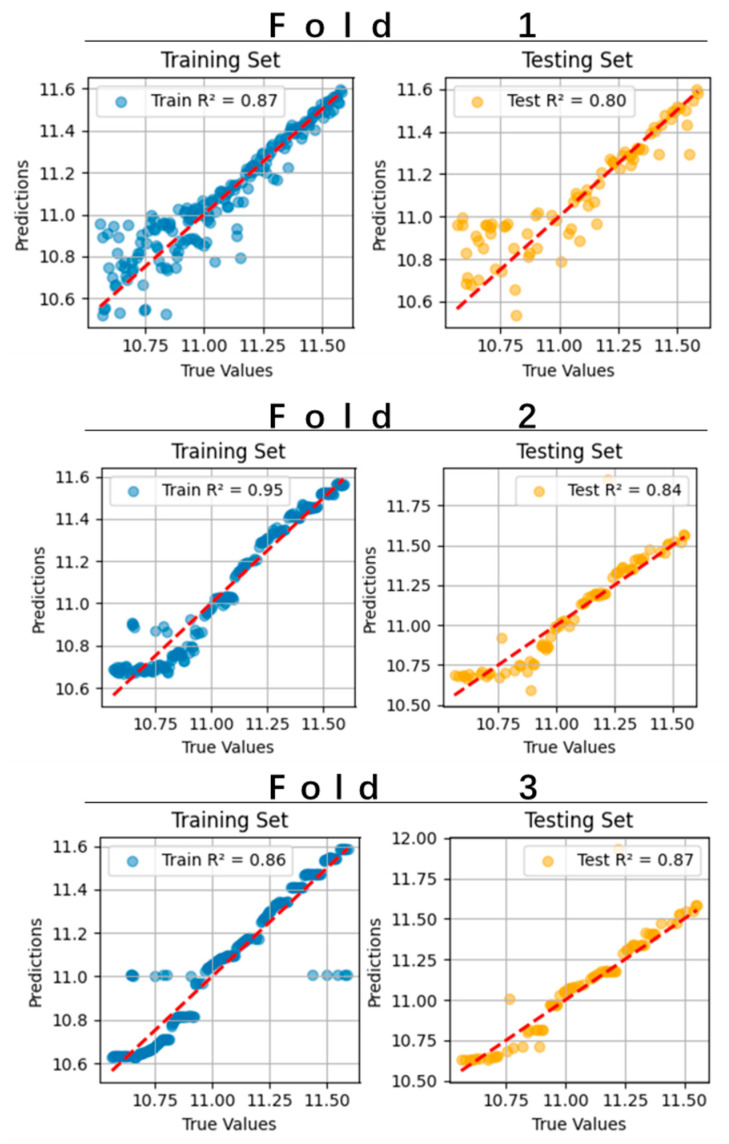
Performance of KAN in three-fold cross-validation for predicting 100 m results.

2. Cross-Validation Metrics:

The pruned model achieved a mean $R^{2}=0.83$ on the test set via three-fold cross-validation (Figure 6), demonstrating favorable predictive accuracy for sprint performance.

3. Symbolic Formula Derivation:

The final equation mapping neuromuscular features to sprint velocity (m/s) was derived as follows:

$$y=11.085-0.0017x_{1}-0.0002x_{3}-0.0016x_{5}-0.0036x_{6}+0.2996x_{7}+0.0005x_{8}-0.0006x_{9}+1.168\times 10^{-5}e^{2.6409x_{2}}+3.561\times 10^{-6}e^{2.6896x_{4}}$$

4. Variable Definitions:
  ○ $x_{1}$−$x_{3}$: α/β/γ-band coherence of the RA-BF muscle pair.
  ○ $x_{4}$−$x_{6}$: α/β/γ-band coherence of the TA-GL muscle pair.
  ○ $x_{7}$−$x_{9}$: Time–frequency features (iEMG, RMS, MF) of the rectus femoris.

## 4. Discussion

This study is the first to reveal that transcranial direct current stimulation (tDCS) applied to the primary motor cortex (M1 region) can enhance athletic performance in the 30–60 m speed maintenance phase of sprinters by increasing intermuscular coherence (IMC) in the gamma band. This finding provides important evidence for understanding the neural mechanisms underlying the optimization of sprint performance through neurostimulation techniques. The results show that tDCS intervention in the M1 region specifically improves performance during the speed maintenance phase while significantly increasing neural synchrony in the lower-limb antagonist muscle groups (rectus femoris–biceps femoris and tibialis anterior–gastrocnemius) in the gamma band. This finding is highly consistent with recent research on the role of gamma-band oscillations in motor control [11]. Previous studies have shown that neural synchrony in the gamma band (30–50 Hz) is closely related to the regulation of excitability in the motor cortex, particularly playing a key role in rapid alternating movements requiring precise temporal control [12]. Our findings further confirm that enhancing excitability in the motor cortex via tDCS can optimize the synchrony of descending motor commands, thereby improving the coordination efficiency of antagonist muscle groups during high-speed running. Notably, this improvement exhibits distinct phase specificity, with significant effects observed only during the speed maintenance phase (30–60 m), while no statistically significant effects were observed during the acceleration phase (0–30 m) or the speed endurance phase (60–100 m). This phenomenon may be related to differences in neural control characteristics across different running phases. During the speed maintenance phase, athletes must maintain stable stride frequency and stride length, which imposes higher demands on the temporal coordination of the neuromuscular system [13]. The acceleration phase relies more on explosive power, while the endurance phase involves more metabolic factors, both of which may weaken the tDCS-induced improvement in neural synchrony [14].

Additionally, the study found that tDCS has a significant selective effect on enhancing gamma-band intermuscular coherence (IMC): a significant increase in gamma-IMC was observed only in the speed maintenance phase (30–60 m) for the RA-BF and TA-GL muscle pairs, while no similar effects were observed in other running phases or in the alpha/beta-frequency bands. This finding provides important clues for understanding the neural oscillation mechanisms underlying the optimization of motor performance through neurostimulation techniques [15]. The results are highly consistent with recent theoretical hypotheses regarding the role of gamma oscillations in motor control. Previous studies have shown that gamma-band neural synchrony (30–50 Hz) is particularly associated with precise temporal control of distal muscles by the motor cortex [16]. Our findings are the first in human motor performance research to confirm that γ-band IMC enhanced by non-invasive brain stimulation can indeed translate into improved functional motor output. Notably, this enhancement of γ-IMC was observed only during the speed maintenance phase, potentially reflecting the higher demands for antagonist muscle coordination during this phase—requiring precise activation timing of the quadriceps and hamstrings during high-speed swinging movements [17]. Compared to previous tDCS studies, the breakthrough finding of this study lies in revealing the dual specificity of the intervention effect: it exhibits both spatial specificity and frequency specificity [18]. This result challenges the traditional view that tDCS broadly enhances cortical excitability, suggesting that its effects may be mediated through specific frequency neural oscillation pathways. Notably, although numerical changes in γ-IMC were observed during the 60–100 m phase, these changes did not reach statistical significance, potentially reflecting alterations in neural oscillation patterns under fatigued conditions, consistent with reports that fatigue reduces cortical muscle coherence [19]. The findings of this study have important implications for sprint training. First, it establishes γ-IMC as a new indicator for assessing neuromuscular coordination, which can be applied to athlete selection and training monitoring in the future. Second, the study suggests that the timing of tDCS intervention should be aligned with specific competition stages, with the greatest benefits potentially achieved during the speed maintenance phase.

This study innovatively applied the KAN to construct a quantitative predictive model linking electromyographic (EMG) features to sprint performance, achieving the first explicit mathematical mapping between neuromuscular coordination parameters and athletic performance. The study found that the optimized KAN model can predict sprint performance effectively (R^2^ = 0.83), and its unique symbolic formula output reveals the differentiated contribution patterns of gamma-band intermuscular coherence and time-domain electromyographic features to athletic performance. This methodological breakthrough provides a new paradigm for modeling complex systems in the field of sports science.

The optimized KAN model offers a fundamentally distinct approach through its unique symbolic derivation capability, converting hidden-layer weights into interpretable mathematical expressions. This capacity for explicit formula generation—unavailable in conventional neural networks or standard machine learning models—directly addresses the stringent interpretability requirements of motor neuroscience research [20], forming the primary distinction emphasized in this study. The exponential function form of the model’s final output is particularly noteworthy, aligning with recent findings on the nonlinear characteristics of the neuromuscular system. Existing theories suggest that the motor cortex exhibits a threshold effect in driving muscle activity, with performance experiencing exponential improvement when neural synchrony reaches a specific level [21]. Our model validates this hypothesis from a data perspective, indicating that gamma-band coherence (x_2_ and x_4_) exerts a critical influence on performance through exponential terms. Compared to conventional linear regression models, KAN captures more complex interactions between electromyographic features and motor performance [22]. For example, the model reveals that the contributions of different frequency-band coherence to performance are not simply additive but exhibit nonlinear coupling relationships [23]. This finding challenges the assumption of “independent effects of each frequency band” in traditional IMC analysis and supports the recently proposed “cross-frequency coupling” theory [24]. The predictive model established in this study has significant application value for athletic training. First, the symbolic formula can be directly used to develop personalized neuromuscular training programs, allowing coaches to predict potential performance improvement based on athletes’ specific muscle group IMC characteristics. It also opens new directions for the application of artificial intelligence technology in sports.

Limitations of the study: This study has several limitations that should be acknowledged. First, the tDCS protocol employed a fixed current density (0.08 mA/cm^2^) without accounting for individual differences in skull thickness, scalp impedance, or cortical excitability, which may have resulted in suboptimal stimulation effects for some participants. Second, while the sham condition (ramp-up to 0.5 mA before discontinuation) is a standard approach, it may not fully mask the sensation of stimulation, potentially introducing placebo effects or participant expectations that could confound the results. Third, environmental factors such as temperature, humidity, and air resistance were not controlled during sprint performance tests, possibly adding variability to neuromuscular and performance measurements. These limitations highlight the need for future research to explore individualized stimulation parameters, improved sham protocols, and standardized environmental controls to enhance the reliability and generalizability of findings.

## 5. Conclusions

This study confirms that transcranial direct current stimulation (tDCS) applied to the primary motor cortex can specifically enhance gamma-band muscle inter-phase coherence during the speed maintenance phase in sprinters, thereby improving athletic performance. This finding reveals the mechanism by which neurostimulation technology improves motor output by optimizing neuromuscular synchrony, providing a theoretical basis for precise intervention. Additionally, the KAN model developed in this study successfully established a quantitative relationship between electromyographic features and athletic performance. Its interpretable mathematical expression not only validates the central role of gamma oscillations in motor control but also provides the field of sports science with a novel analytical tool that combines predictive accuracy with physiological interpretability. These findings collectively advance the scientific application of neuromodulation techniques in competitive sports, opening new avenues for athlete training monitoring and performance enhancement.

## Figures and Tables

**Table 2:** Comparison of 100 m performance test under different conditions.

	Sham	Motor Cortex tDCS	DLPFC tDCS	F	*p*
0–30 m (m/s)	9.26 ± 0.44	9.35 ± 0.40	8.97 ± 0.49	2.17	0.134
30–60 m (m/s)	11.12 ± 0.60	11.59 ± 0.67 ^a^	11.32 ± 0.59	4.62	0.019
60–100 m (m/s)	10.08 ± 0.32	9.88 ± 0.34	9.85 ± 0.40	2.60	0.092

Note: ^a^ represents a significant difference compared to the sham. DLPFC: dorsolateral prefrontal cortex.

**Table 3:** Comparison of intermuscular coherence under different conditions.

			Sham	Motor Cortex tDCS	DLPFC tDCS	*p*
0–30 m	RA-BF	α	0.20 ± 0.06	0.25 ± 0.07	0.18 ± 0.06	0.307
		β	0.41 ± 0.18	0.48 ± 0.20	0.39 ± 0.17	0.603
		γ	0.88 ± 0.33	0.95 ± 0.35	0.82 ± 0.30	0.764
	TA-GL	α	0.12 ± 0.07	0.15 ± 0.08	0.10 ± 0.06	0.631
		β	0.25 ± 0.14	0.30 ± 0.15	0.22 ± 0.13	0.356
		γ	0.68 ± 0.43	0.75 ± 0.45	0.65 ± 0.40	0.739
30–60 m	RA-BF	α	0.24 ± 0.10	0.32 ± 0.11	0.20 ± 0.09	0.361
		β	0.44 ± 0.21	0.55 ± 0.23	0.40 ± 0.20	0.208
		γ	0.55 ± 0.23	0.70 ± 0.25 ^a^	0.50 ± 0.22 ^b^	0.005
	TA-GL	α	0.09 ± 0.06	0.12 ± 0.07	0.08 ± 0.05	0.568
		β	0.08 ± 0.03	0.11 ± 0.04	0.07 ± 0.03	0.065
		γ	0.34 ± 0.12	0.45 ± 0.14 ^a^	0.30 ± 0.11 ^b^	0.025
60–100 m	RA-BF	α	0.23 ± 0.17	0.20 ± 0.16	0.25 ± 0.18	0.952
		β	0.53 ± 0.29	0.60 ± 0.32	0.50 ± 0.28	0.594
		γ	0.15 ± 0.07	0.12 ± 0.06	0.16 ± 0.07	0.828
	TA-GL	α	0.22 ± 0.15	0.25 ± 0.16	0.20 ± 0.14	0.549
		β	0.51 ± 0.29	0.58 ± 0.31	0.48 ± 0.27	0.535
		γ	0.98 ± 0.45	1.10 ± 0.50	0.90 ± 0.42	0.212

Note: ^a^ represents a significant difference compared to the sham. ^b^ represents a significant difference compared to the motor cortex tDCS. RA: rectus femoris; BF: biceps femoris; GL: lateral gastrocnemius; TA: tibialis anterior; DLPFC: dorsolateral prefrontal cortex.

## Data Availability

All data from this study are in the manuscript; please contact the corresponding author if you need anything else.
